# Circulating miRNA Biomarkers for Alzheimer's Disease

**DOI:** 10.1371/journal.pone.0069807

**Published:** 2013-07-29

**Authors:** Pavan Kumar, Zoltan Dezso, Crystal MacKenzie, Judy Oestreicher, Sergei Agoulnik, Michael Byrne, Francois Bernier, Mamoru Yanagimachi, Ken Aoshima, Yoshiya Oda

**Affiliations:** 1 Eisai Inc, Biomarkers and Personalized Medicine Core Function Unit, Eisai Product Creation Systems, Andover, Massachusetts, United States of America; 2 Eisai Co., Ltd. Biomarkers and Personalized Medicine Core Function Unit, Eisai Product Creation Systems, Tsukuba Research Laboratories, Ibaraki, Japan; University of Melbourne, Australia

## Abstract

A minimally invasive diagnostic assay for early detection of Alzheimer's disease (AD) is required to select optimal patient groups in clinical trials, monitor disease progression and response to treatment, and to better plan patient clinical care. Blood is an attractive source for biomarkers due to minimal discomfort to the patient, encouraging greater compliance in clinical trials and frequent testing. MiRNAs belong to the class of non-coding regulatory RNA molecules of ∼22 nt length and are now recognized to regulate ∼60% of all known genes through post-transcriptional gene silencing (RNAi). They have potential as useful biomarkers for clinical use because of their stability and ease of detection in many tissues, especially blood. Circulating profiles of miRNAs have been shown to discriminate different tumor types, indicate staging and progression of the disease and to be useful as prognostic markers. Recently their role in neurodegenerative diseases, both as diagnostic biomarkers as well as explaining basic disease etiology has come into focus. Here we report the discovery and validation of a unique circulating 7-miRNA signature (hsa-let-7d-5p, hsa-let-7g-5p, hsa-miR-15b-5p, hsa-miR-142-3p, hsa-miR-191-5p, hsa-miR-301a-3p and hsa-miR-545-3p) in plasma, which could distinguish AD patients from normal controls (NC) with >95% accuracy (AUC of 0.953). There was a >2 fold difference for all signature miRNAs between the AD and NC samples, with p-values<0.05. Pathway analysis, taking into account enriched target mRNAs for these signature miRNAs was also carried out, suggesting that the disturbance of multiple enzymatic pathways including lipid metabolism could play a role in AD etiology.

## Introduction

Alzheimer's disease (AD) is a progressive neurodegenerative disease manifested by dementia typically observed in the elderly. Symptoms include disorientation, loss of memory, visual-spatial skills, and psychiatric symptoms. Approximately 24 million people worldwide have dementia, of which the majority (∼60%) is due to AD [1]. The neuropathology of AD is characterized by the presence of amyloid plaques, neurofibrillary tangles, synaptic loss and selective neuronal cell death in the brain [2]. Amyloid plaques result from abnormal levels of extracellular amyloid beta (Aβ) peptide, which are products of sequential enzymatic cleavages of ß-amyloid precursor protein (APP) by ß- secretase (BACE1) and γ-secretase. Neurofibrillary tangles on the other hand are associated with the presence of intracellular hyper-phosphorylated tau protein. Compared with normal tau, which contains two to three phosphate groups, the p-tau contains 5–9 phosphate groups per protein and inhibits the normal tau-promoted microtubule assembly [3].

AD is currently diagnosed using a combination of clinical criteria [4], which includes a neurological exam, mental status tests, and brain imaging [5]. An AD diagnosis is also sometimes reached by eliminating other causes of dementia. Based on these criteria, an accurate diagnosis can be difficult, especially for patients having mild or early-stage AD. Accordingly, needs exists for biomarkers that are indicative of AD and may be used for earlier diagnosis on living patients. Earlier diagnosis of AD and subsequent intervention is also thought to be socially desirable in terms of increasing economic efficiency, in addition to significantly reducing health care costs by delaying entry of AD patients into nursing homes for long term care [6]. However, the need for biomarkers in neurodegenerative diseases is not limited to diagnostic purposes only. The testing and ultimate implementation of emerging therapies will also require identification of affected and “at-risk” individuals to target them for clinical trials. AD patients are known to have neuropathology in their brains for over 10 to 20 years before any symptoms occur. With ongoing research to develop new AD treatments, an increasing need to establish an early diagnosis of AD could become important [7]. So in addition to traditional diagnostic value, biomarkers are now being investigated for use in patient stratification, following patient response to treatment and making regiment changes if a drug is not providing the desired benefit.

Currently, bio-fluid derived markers and neuroimaging techniques are being explored as possible biomarkers for early-stage and pre-clinical AD diagnosis, because it is in these initial stages that disease-modifying therapies are likely to have the greatest chance of preserving normal brain function [8]. Among these, cerebrospinal fluid (CSF) is a very attractive and potent source of biomarkers for brain-related conditions, since it could serve as surrogate readout of the brain condition, in terms of both metabolic and biochemical profiles. In AD, CSF concentrations of soluble Aβ (1–42) are reduced by 40–50% compared to age-matched healthy controls [9]. Recently, multiple reports also highlighted the value of looking at the ratio of Aβ40 and Aβ42 peptides in the CSF, which could help serve as an indicator of AD [10] [11] [12]. Another well accepted indicator for AD is the level of phopho-tau (p-tau) in CSF [13], which is now considered as an *in vivo* surrogate biomarker of neurofibrillary pathology in AD. To further strengthen this hypothesis, there have now been reports which also found positive correlations between ratio of Aβ40 and Aβ42 peptides and p-tau in CSF [14]. Unfortunately, both tau and p-tau cannot be used to correctly identify AD since changes of these molecules have also been implicated in other neurodegenerative diseases [15].

Another source of biomarkers that has been the focus of attention of researchers, especially in cancer biology and cardiovascular diseases is blood [16] [17] [18]. An advantage of using blood-based markers is the ease and possible frequency of collection of sample from patients. In contrast, CSF biomarkers would require invasive and delicate lumbar punctures, which can be performed only by trained physicians. There have been multiple reports of plasma/serum markers identified, which could potentially distinguish various neurodegenerative conditions including AD [19], Parkinson's disease (PD) [20] and schizophrenia [21]. For example, in PD, it was recently reported that deregulation of the RNA splicing factor SRRM2 in peripheral blood cells correlated with PD, and not with other neurodegenerative diseases [22]. Another area of interest is the auto-immune response to amyloidogenic proteins associated with diseases and their applications in therapeutic treatments such as vaccination with amyloid antigens and antibodies in PD, AD and potentially other neurodegeneration ailments. It was recently reported that α-synuclein reactive antibodies in the blood sera could serve as diagnostic markers for PD [23] . α-synuclein is classified as a natural amyloidogenic protein and conversion from its soluble cytosolic state to an aggregated insoluble form is one of the key events in the pathogenesis of PD. In AD, six plasma biomarkers including AAT (α1-antitrypsin) and ApoJ were identified to be up-regulated with disease [24]. AAT was further validated by ELISA and it was also found to be present in neurofibrillary tangles and senile plaques [25].

In addition to the existing proteomic, metabolomics and genomic class of circulating biomarker molecules, miRNA is another novel class of circulating molecules that has gained significant attention. MiRNAs belong to the class of non-coding regulatory RNA molecules of ∼22 nt length that modify gene expression at the post-transcriptional level by primarily binding to the 3′ un-translated region (UTRs) of their target messenger RNAs [26] [27] [28]. It is estimated that 1–4% genes in the human genome encode for miRNAs and a single miRNA can regulate as many as 200 mRNAs [29]. There is increasing evidence suggesting that miRNAs play critical roles in many key biological processes, such as cell growth, tissue differentiation, cell proliferation, embryonic development, and apoptosis [30]. Recently it had been shown, that not only are they active in the cell in which they are produced, but they can be exported out of their production “host” cell, and cause down regulation of target mRNAs in a distant “target” cell [31] [32]. It is this particular property of miRNAs of being abundantly found in stable functional condition in all biological circulating fluids including plasma, urine, tears, saliva and CSF [33], which makes them promising candidates as biomarkers. They are found enclosed in exosomes and other vesicular structures [34] [35], and even freely circulating in fluids, protected by RNA binding proteins including NPM1 [36], HDL [31], [37] or Argonaute2 [38] [39]. Circulating profiles of miRNAs have been shown to discriminate different tumor types, [40] [41],[42] [43] [44] indicate staging and progression of the disease [45] and serve as prognostic markers [46] [47].

Although, most of the initial findings highlighting diagnostic potential of miRNAs were in the field of oncology, significant work has recently been published investigating the role of miRNAs in different neurodegeneration diseases. Their role is not limited to a diagnostic viewpoint, but also provides a further understanding of disease etiology. In schizophrenia, a seven-miRNA signature (hsa-miR-34a, hsa-miR-449a, hsa-miR-564, hsa-miR-432, hsa-miR-548d, hsa-miR-572 and hsa-miR-652) was identified in mononuclear leukocytes, which were found to be correlated with patients' negative symptoms, neurocognitive performance scores, and event-related potentials [48]. From a disease etiology point of view, functional role of hsa-miRNA-219 was illustrated by evidence that it modulated NMDA receptor-mediated neurobehavioral dysfunction by targeting CaMKIIgamma, a component of the NMDA receptor signaling cascade in the pre frontal cortex (PFC) of mice [49]. In Alzheimer's, miRNA profiling experiments have resulted in identification of disease-specific miRNAs that have been validated in two or more independent studies [50]. For example, hsa-miR-106, hsa-miR-153 and hsa-miR-101 have been shown to target APP [51] [52] [53] [54] , while hsa-miRNA-29 and hsa-miR-107 have been shown to target BACE1, linking them to regulation of amyloid production in AD brains [55]. In view of these studies, researchers have focused attention on these miRNAs to determine if differential levels are found in circulating fluids, for example blood or CSF. Hsa-miR-29a/b amongst others was an example in which, a miRNA discovered to be linked to AD brain pathology was also found to be down-regulated in serum of AD patients [56].

In contrast to the above approaches, there is an unbiased global approach of miRNA profiling. This is not limited to miRNAs that have been shown to have a direct connection with disease, or enriched in the tissue of interest. This approach allows researchers to profile global miRNA expression levels using relatively small amount of purified RNA in a specific and sensitive manner. It provides an opportunity to study the underlying mechanisms involved in AD etiology, which might not have been previous associated with the disease. Illustrating this approach, miRNAs were identified in CSF and brain tissues that were found to be differentially regulated between AD and age and sex-matched normal control (NC) populations. Of particular interest were expression patterns of hsa-miR-9 and hsa-miR-132, which based on their known (and predicted targets) were linked to the alterations in stem cell commitment, neuronal differentiation and actin remodeling [57]. Furthermore, the targets of miRNAs identified included genes associated with pathways such as brain insulin signaling and regulation of oxidative stress in the brain, that were previously not particularly well-associated with AD. However, there was no obvious relationship observed between altered miRNA profiles in CSF and specific brain regions thought to be most affected by the disease. In fact, most of the altered miRNAs in the CSF were determined to be of immune cell origin, like miR-146b, which is implicated in innate immunity response [58]. It was found to be down regulated in AD patients, indicating an activated immune response.

In this study, we decided to take a global profiling approach and measured circulating plasma miRNA expression from a total 11 AD and 20 NC patients using the Nanostring platform [59]. We measured a total of 654 human miRNAs, out of which 12 miRNAs had differential expression in AD samples. Out of these, down-regulation of 7 miRNAs (hsa-let-7d-5p, hsa-let-7g-5p, hsa-miR-15b-5p, hsa-miR-142-3p, hsa-miR-191-5p, hsa-miR-301a-3p and hsa-miR-545-3p) was confirmed using singleplex qPCR. This signature was then further validated using an independent cohort of 20 AD and 17 NC samples, resulting in positive correlations across the two independent cohorts with up to 95% prediction accuracies. Using experimentally validated and predicted miRNA targets, we identified novel and some established Alzheimer related biological pathways, which could potentially lead to novel targets for drugs and shed some light on AD etiology.

## Materials and Methods

Plasma sample acquisition: Human plasma samples were purchased from Precision Med (Solano Beach, CA-USA). Cohort 1 consisted of 11 Alzheimer (AD), 9 MCI and 20 NC patient samples. Cohort 2 consisted of 20 AD and 17 NC patient samples. Age, sex, diagnosis and MMSE scores are provided in Tables S1 & S2 in File SI.

Total RNA extraction: Total RNA was extracted using miRvana PARIS kit (Life Technologies) with modifications as described [60]. Briefly, 1 mL of human plasma was added to an equal amount of 2× denaturing buffer and then spiked with 10 µl of 0.05 µM synthetic Ath-159a (Table S3 in File SI) and 40 pM of NegA (UUGUGGCGAGCGGAAUGGAAU) (synthetic miRNAs used for normalization and extraction efficiency control for the Nanostring assay). Phenol extraction was performed twice, and total RNA was finally eluted in 70 µl of dH_2_O following recommended protocol.

High-throughput expression profiling of miRNAs: 45 µl of purified total RNA was concentrated down to 9 µl and used as template for the nCounter miRNA expression assay v1 (Nanostring Technology, Seattle, WA-USA). The sample preparation was set up as recommended (Nanostring; C-0009-02) using 3 µl of concentrated total RNA as starting amount for all samples in triplicate. The reactions were set up for an overnight hybridization for 16 hours at 65°C. The following day, the samples were processed through the nCounter Prep Station (v.20081003) as recommended followed by processing through the nCounter Digital Analyzer (v.20081009). The analyzer resolution was set at 600 FOVs. Data was downloaded and analyzed on Excel. Briefly, the data were first normalized for lane-to-lane variation using the provided positive assay controls. This was followed by a global mean normalization by using the counts of the highest 100 miRNA expressers. Each normalized value was then checked to ensure that it was at least 2 SDs higher than the average of background signal recorded for that lane. Any value below that was converted to zero. Fold change values were then calculated by taking the average of all AD/MCI and NC sample expression values for individual miRNAs. Candidate miRNAs were chosen which at least had a 1.5 fold difference between average AD/MCI and NC samples and had average normalized counts >150.

Validation of candidate biomarkers using TaqMan qPCR: Candidate miRNA biomarkers identified using the high-throughput Nanostring platform were validated by using stem-loop TaqMan RT-qPCR miRNA assays (Life Technologies). Briefly, a RT primer pool was created with specific miRNA RT primers (hsa-miR-301a-3p, hsa-miR-1975a, hsa-miR-191-5p, hsa-miR-15b-5p, hsa-let-7g-5p, hsa-let-7d-5p, hsa-miR-545-3p, hsa-miR-1274a, hsa-miR-142-3p, hsa-miR-600, hsa-miR-323b-5p-5p, hsa-miR-563, hsa-miR-106a-5p and ath-159a) at a final concentration of 0.05× in 1× TE. A 15 µl RT reaction was set up containing 6 µl of the RT primer pool, 0.3 µL dNTPs (100 mM), 3 µl of Multiscribe RT (50U/µl), 1.5 µl of the 10× Reverse Transcription 4366596). Three µl of total RNA (1∶10 dilution) was added as template for each sample and the reaction was incubated on ice for 5 min followed by 30 min at 16°C, 30 min at 42°C and 5 min at 85°C for enzyme inactivation. The reaction was then stored at 4°C. A second pool of pre-amplification primers was then created with each PCR primer probe (20×) for the same assays mixed at a final concentration of 0.2× in 1× TE. A pre-amplification reaction was set up containing 2× Pre-amplification master mix (Life Technologies), 3.75 µl of the custom pre-amplification primer pool and dH_2_O (making up the reaction volume to 22.5 µl). 2.5 µl of the RT product was then added and cycled through the pre-amplification program [95°C for 10 min, 55°C for 2 min, and 72°C for 2 min followed by 12 cycles of 95°C for 15 sec and 60°C for 4 min]. This was followed by 99.9°C incubation for 10 min, and the reaction was stored at 4°C. The reaction was diluted by adding 175 µl of 0.1× TE (pH 8.0) and mixed by inversion. Two µl of the pre-amplification product was then used for singleplex standard TaqMan qPCR reactions (in duplicate) following standard protocol (Life Technologies; P/N 4364031-Rev D) on an ABI7500 instrument. The ΔΔCt method was used for the analysis with the geometric mean of both hsa-miR-106a-5p and ath-159a used for sample-to-sample normalization, and the average relative Ct values of NC patients being used to calibrate all the individual values. Linear fold changes were then calculated and plotted on scatter plots using Prism (GraphPad Prism Software, San Diego, USA).

Signature Generation and Prediction: The miRNAs identified and validated as having significantly different expression between AD and NC samples (fold change>1.5, p value<0.05) based on Cohort 1 (11 AD and 20 NC samples) were selected for predictive model building. The expression values of 7 miRNAs (hsa-let-7d-5p, hsa-miR-191-5p, hsa-miR-301a-3p, hsa-miR-545-3p, hsa-let-7g-5p, hsa-miR-15b-5p, hsa-miR-142-3p) based on the Taqman qPCR values of Cohort 1 were used to build linear classifier to separate AD from NC samples. All possible non-zero subsets of the 7 miRNA (127 signatures) were used for the linear discriminatory analysis (LDA) and the performance of the predictive model was evaluated based on the classification results of the samples from Cohort 2 (20 AD and 17 NC). The performance of the predictive model was evaluated based on accuracy, specificity, sensitivity and area under curve (AUC) numbers.

For the LDA the MASS package was used in R statistical environment [61]. The AUC numbers were also calculated in R using the package “verification”. (http://cran.r-project.org/web/packages/verification/index.html) [62].

Pathway analysis: The IPA Ingenuity software was used to identify pathways enriched in the 7-miRNA targets. We applied IPA's miRNA Target Filter to identify both experimentally validated and predicted mRNA targets. Next we identified those neurology related pathways, which were significantly enriched in genes targeted by at least 2 signature miRNAs. Both experimentally validated interactions and predictions characterized as “high” confidence were considered based on TargetScan. In our second approach we applied an unbiased pathway enrichment analysis without any filter on pathway functions. We considered pathways enriched in genes targeted by at least 2 miRNA with “high” or “moderate” prediction confidence for the analysis. Given that majority of the miRNA work thus far has been in the field of Oncology, experimentally validated miRNA target genes were excluded.

## Results and Discussion

The aim of this study was to generate miRNA profiles from the plasma fraction of human blood, and determine if there were significant differences in both miRNA content, and expression levels of individual miRNAs between patients diagnosed with Alzheimer's (AD), mild cognitive impairment (MCI) or normal control (NC) patients. Plasma samples were obtained from Precision Med (Solano Beach, USA). Cohort 1 contained 11 AD, 9 MCI and 20 NC samples, while cohort 2 containing 20 AD and 17 age matched NC samples. Total RNA was extracted and spike-in synthetic miRNAs (Ath-159a and Neg-A) were added to control for extraction efficiency as described in the methods section. Each sample was then used for high-throughput expression profiling using the nCounter miRNA expression assay (Nanostring; Seattle,USA) in triplicate as described. The global-mean of top-100 miRNA expressers in the samples tested was used to content normalize the miRNA expression across all samples as described previously [63] [64]. Post normalization and back-ground correction, the final linear counts were averaged for both AD/MCI and NC samples and fold change values were calculated. There were 227 miRNAs identified which at least had an average expression count >100 (considered significantly expressed over background in this study). Out of the 227 miRNAs, we identified 12 miRNAs, which had at least a 1.5 fold difference, and had an average of >150 normalized linear counts (Fig. S1). These included hsa-let-7d-5p, hsa-let-7g-5p, hsa-miR-15b-5p, hsa-miR-142-3p, hsa-miR-191-5p, hsa-miR-301a-3p, hsa-miR-323b-5p, hsa-miR-545-3p, hsa-miR-563, hsa-miR-600, hsa-miR-1274a and hsa-miR-1975. All sequence and miRBase accession numbers (v19) are provided in Table S3 (File SI).

For further validation, we focused on only AD and NC patients, and proceeded to validate these candidate biomarker miRNAs using the Stem Loop TaqMan RT-qPCR miRNA assay (Life Technologies). A 1∶10 dilution of total RNA was made, and run through the TaqMan assay and analyzed as described in the methods section. Due to lack of a consensus normalization control for circulating miRNAs, we took an unbiased approach and focused on miRNAs characterized with the least variation in ranks among the samples as determined by the nCounter assay. We applied an algorithm with several iterative steps to determine the top “rank invariant” miRNA as described [65] and hsa-miR-106a was identified. Hence we used the geometric mean of the spike-in (ath-159a) and endogenous miRNA (hsa-miR-106a) for normalization of our validation data obtained by TaqMan qPCR to take care of both the extraction efficiency variation as well as endogenous miRNA differences. In order to further confirm that our choice of endogenous miRNA had no effect on the validity of the signature, we re-analyzed the validation data set using only the spike-in (ath-159a) for normalization across all samples. We achieved consistent down-regulation of the identified signature miRNAs with p-values<0.005 (Table S4 in File SI).

**Figure 1:**
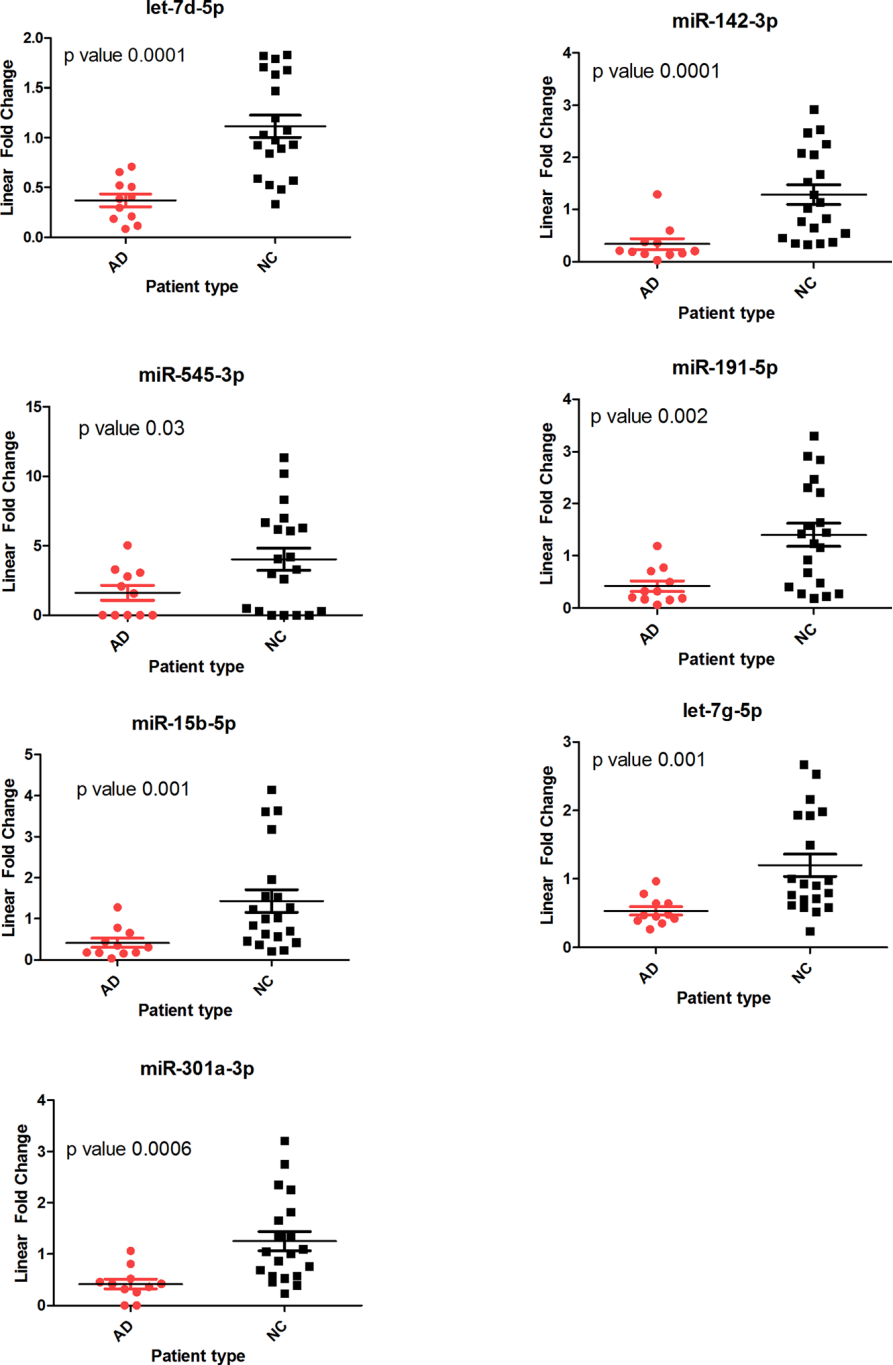
Scatter plots of validated miRNAs differentiating Alzheimer and NC samples in Cohort 1. Total RNA extracted from plasma samples was used for validating miRNA expression values using singleplex TaqMan assays. Ath-159a (spike-in) and hsa-miR-106a-5p (endogenous) was used for normalization. All values were then normalized relative to the average of the 20 NC samples and plotted on the Y-axis.

Out of the 12 miRNAs identified using the nCounter assay; we could not confirm the differential expression of 5 miRNAs using the described methods. These included hsa-miR-323b-5p, hsa-miR-563, hsa-miR-600, has-miR-1274a and hsa-miR-1975. The remaining 7 miRNAs showed significant differences between AD and NC patient cohorts (Fig. 1 & Table 1). For example, hsa-miR-191-5p was down regulated in AD samples by 3 fold according to Nanostring analysis, while it was found to be down-regulated by ∼4 fold by TaqMan analysis. This close trend was observed for hsa-miR-15b-5p, hsa-let-7d-5p, hsa-let-7g-5p and hsa-miR-142-3p. All fold change values had a p-value<0.05. We could not however replicate the up-regulation observed for hsa-miR-301a-3p and hsa-miR-545-3p in AD/MCI patient samples using the nCounter assay, and in fact we observed significant down-regulation of these two miRNAs in the TaqMan analysis. These two miRNAs were originally chosen to be validated because they were found to be up-regulated in the AD/MCI patient samples as opposed to the rest of the miRNAs, which were down-regulated. But if you consider only the AD patient samples, then the difference between AD and NC samples was not-significant in the nCounter assay (Table 1). It is also noteworthy that all candidate miRNAs, which could not be confirmed by TaqMan assays, had normalized linear counts (from Nanostring) of less than 500. The average count of all the validated miRNAs on the other hand was higher than 3,000. This suggests the importance of setting a higher threshold for future plasma based Nanostring experiments to lessen the false-positive rate.

**Table 1:** Differential expression of validated signature miRNAs for AD and NC samples.

miRNA name	Nanostring Cohort 1		TaqMan Cohort 1		TaqMan Cohort 2	
	Fold Change	P value	Fold Change	P value	Fold Change	P value
hsa-let-7d-5p	1.724	0.0266	3.01	0.0001	3.03	<0.0001
hsa-let-7g-5p	1.786	0.0291	2.26	0.001	2.62	<0.0001
hsa-miR-15b-5p	2.759	0.0128	3.45	0.001	3.65	<0.0001
hsa-miR-142-3p	2.473	0.0283	3.84	0.0001	5.04	<0.0001
hsa-miR-191-5p	2.924	0.0054	3.38	0.002	5.15	<0.0001
hsa-miR-301a-3p	0.833	ns	2.98	0.0006	1.35	0.07
hsa-miR-545-3p	0.625	ns	2.49	0.03	2.37	0.01

**Figure 2:**
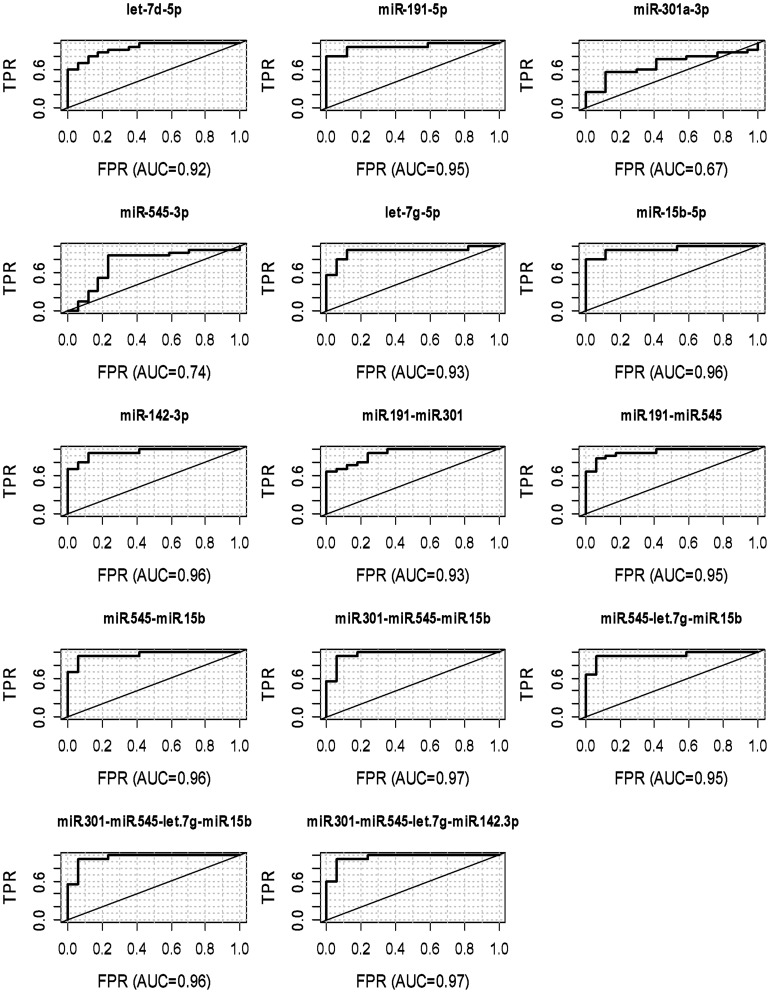
The receiver-operating characteristic (ROC) plots for the miRNA signature. The true positive rate (TPR) is plotted as a function of false positive rate (FPR) for the 7 miRNAs individually (upper panel) and for selected combination of them (lower panel). We used the linear discriminant analysis (LDA) to build a model on the training data (11 AD and 17 controls) to predict the AD status of 37 patients (20 AD and 17 controls). These miRNA combinations are all characterized with high area under the curve (AUC) values (>0.93).

The relative Ct values (observed Ct values of individual miRNAs minus the geometric mean of ath-159a and hsa-miR-106a-5p Ct values) for all the samples and candidate miRNAs (hsa-let-7d-5p, hsa-let-7g-5p, hsa-miR-15b-5p, hsa-miR-142-3p, hsa-miR-191-5p, hsa-miR-301a-3p and hsa-miR-545-3p) were then used to build a signature model as described in the methods section. This signature was then used to predict the disease status of cohort 2 patients (20 AD, 17 control). Total RNA extraction was carried out, followed by TaqMan RT-qPCR analysis. We achieved significant correlation with average fold changes between the two cohorts for the signature miRNAs being closely replicated (Table 1 and Fig. S2). The relative Ct values (observed Ct values of individual miRNAs minus the geometric mean of Ath-159a and hsa-miR-106a-5p Ct values) for all the samples and candidate miRNAs (hsa-let-7d-5p, hsa-let-7g-5p, hsa-miR-15b-5p, hsa-miR-142-3p, hsa-miR-191-5p, hsa-miR-301a-3p and hsa-miR-545-3p) from cohort 2 were then used as input for the signature miRNA prediction algorithm, which was developed based on Cohort 1 data as described above. The performance of the models based on both individual miRNAs as well as selected combinations of the biomarker miRNAs are shown in Fig. 2. The over-all signature accuracy was calculated as the fraction of misclassified samples (AD or NC). The sensitivity characterizes the predictive models ability to identify true positives, which in this study was calculated as the number of correctly identified AD samples divided by the total number of AD samples. On the other hand, the specificity was the number of correctly classified NC samples (true negatives) divided by the total number of NC samples. The AUC was calculated as the area under the receiver operating characteristic (ROC) curve, which is the plot of the sensitivity vs. the false positive rate (1- specificity). The ROC curve was generated by varying the threshold for the prediction probability.

The combination of hsa-miR-545-3p, hsa-let-7g-5p and hsa-miR-15b-5p resulted in the highest specificity (94.1%), sensitivity (95%) and AUC (0.953). The signature combinations ranged from just two miRNAs (e.g. hsa-miR-15b-5p and hsa-miR-545-3p) up to 6 miRNAs (e.g. hsa-miR-191-5p, hsa-miR-301a-3p, hsa-miR-545-3p, hsa-let-7g-5p, hsa-miR-15b-5p and hsa-miR-142-3p). Each signature had different combinations of the candidate miRNA biomarkers and resulted in varying specificity and sensitivity. Selected signatures with specificity >0.9, sensitivity >0.8 and AUC>0.9 are listed in Table S5 (File SI). The signature accuracy for individual miRNAs was lower in comparison to combination signatures. The best stand-alone miRNAs in terms of specificity were hsa-miR-142-3p and hsa-miR-301a-3p, with both having 100% specificity. But hsa-miR-142-3p had better sensitivity (65%) as compared to only 25% sensitivity for hsa-miR-301a-3p. Hsa-let-7g-5p and hsa-miR-191-5p had the best sensitivity at 95%, but only hsa-miR-191-5p had enriched specificity of 76%. Over-all accuracy of hsa-miR-191-5p, hsa-miR-15b-5p and hsa-let-7d-5p was the three best as stand-alone miRNA biomarker candidates.

There have been previous reports published in the literature, which highlighted specific miRNA profiles differentiating patients suffering from various neurodegenerative diseases and healthy individuals. But most studies were focused on the analysis of only those miRNAs, whose expression level changes had been previously identified to be linked to pathology development in the disease organ [6], [50], [51], [52] [55] [56]. With its advantage of the potential biomarker's known connection to disease withstanding, there are a couple of drawbacks to this approach. Due to the nature of circulating fluids being the common reservoir for all secreted molecules from all organs and tissues, the candidate miRNA biomarker could be involved in diseases of various other organs, hence making the correlation harder. Also, higher expression of a miRNA in an affected organ is not necessarily accompanied by an increase in its plasma level, as was previously shown [66] [67]. In a related approach, instead of focusing on the disease etiology, some studies have focused on a selected panel of miRNAs, which were determined to be enriched in brain and neurons [68], thus increasing the likelihood that any changes in plasma or serum is a result of changes in neurons versus other organs. Since MCI and early stages of AD are associated with neurite and synapse destruction, they also included miRNAs known to be present in neurite and synapses [42], [69] [70] and involved in neurite- and synapse-associated processes [71]. They identified differential levels of circulating serum miR-132 and the miR-134 family of miRNAs, which could differentiate MCI from NC population. They also reported that in a separate longitudinal study, these biomarker miRNAs could identify MCI patients at an asymptomatic stage 1–5 years before clinical diagnosis. In the light of recent trial outcomes, this early diagnosis offers promise for better patient stratification and subsequent successful outcomes of Alzheimer drug trials.

In contrast to the above approaches, our approach was more closely aligned to the global miRNA profiling strategy employed by Cogswell et al. [57]. As opposed to CSF samples that cannot be routinely collected from patients, blood is a far more accessible, amenable and less-intrusive source of biomarkers. In support of the hypothesis that plasma biomarkers can act as a surrogate for events in the brain, it was recently reported that a number of plasma proteins were significantly associated with whole brain and hippocampal atrophy in AD [72]. In addition, a panel of signaling proteins in the plasma was also described to predict AD phenotype, suggesting that it is conceivable that a compromised blood-brain barrier in AD and other neurodegenerative disorders, will allow the exchange of molecules between the CNS and the periphery [73] [74].

Further supporting the potential of plasma biomarkers to serve as accurate indicators of neurodegenerative disease; an independent study across 3 centers, including ADNI (Alzheimer's Disease Neuroimaging Initiative) examined over 1000 samples and found four analytes (apoE, B-type natriuretic peptide, C-reactive protein, pancreatic polypeptide) which were altered in clinical MCI/AD across all three independent cohorts. In addition, regression analysis showed CSF Aβ42 levels and t-tau/Aβ42 ratios to correlate with the number of APOE4 alleles and plasma levels of B-type natriuretic peptide and pancreatic polypeptide [75]. Consistent with putative biomarkers identified in CSF, the ratio of plasma Aß1–42/1–40 has also been proposed as a more accurate indicator than each peptide on its own. A low plasma ratio was associated with significantly higher risk of conversion to MCI or AD, whereas the levels of Aß42 or Aß40 on their own did not have any diagnostic value [76].

Recent reports have described the horizontal transfer of biomolecules by secreted extracellular vesicles, which is increasingly becoming established as a general mode of intercellular communication [77]. Exosomes are released by fusion of multivesicular bodies (MVBs) with the plasma membrane and secretion of the intraluminal vesicles (ILVs) into the extracellular space. They are 50–100 nm in diameter and carry specific protein and RNA cargo, including significant number of both precursor and mature miRNAs [78]. Interestingly, neurons and the major types of glia also release vesicles raising the possibility, that this form of inter-cellular communication is a common mechanism in the CNS as well. The fact that a mixed population of such vesicles has also been detected in CSF [79] offers a way in which miRNAs could be transported from neurons and other cell types, crossing the blood brain barrier . Another important study supporting the ability of exosomes to cross the blood brain barrier was recently published, in which researchers engineered mouse dendritic cell derived exosomes to target neurons by the RGV peptide, and achieved ∼60% reduction of BACE1 mRNA and protein using RNAi in the mouse brain [80].

In an effort to understand how our signature miRNAs were indicative of AD, we adopted a pathway analysis approach to identify potential pathways that were enriched for targets of these signature miRNAs. Is it now established that there exists a “many to many” relationship between miRNAs and its target mRNAs. This means that while a single miRNAs can target multiple mRNAs, a single mRNA can also be targeted by multiple miRNAs [81]. The relationship of a single miRNA to potentially target as many as 200 different mRNA targets is well documented [82] [83], but there is also evidence of single mRNAs being targets of multiple miRNAs [84]. To put this complex “many to many” relationship in a biological context, a comprehensive analysis of all miRNA targets suggested that a set of miRNA targets regulated by a single miRNA generally constitutes a biological network of functionally-associated molecules in human cells [85]. This might help to extract some biologically relevant targets amongst the high numbers of “predicted targets” of individual miRNAs and potentially serves as a filter when using pathway analysis tools to understand the functional pathways which are affected by miRNA profile changes.

For this study, we focused on only those mRNAs that were targeted by at least 2 or more of the signature miRNAs. We first compiled a list of top canonical pathways using Ingenuity Pathway Analysis platform (IPA), which were functionally enriched with gene targets of the 7 signature miRNAs (Table 2). This functional enrichment was further overlapped to select pathways that were identified as neurology associated pathways and contained genes, which were targeted by at least 2 signature miRNAs. Among the top 10 pathways, we identified key pathways that had biological links to Alzheimer's biology. This included pathways like axonal guidance signaling, ephrin receptor signaling [86], actin cytoskeleton signaling [87], clathrin-mediated endocytosis signaling [88] and rhoA signaling [89]. These pathways, although diverse show connections with AD pathology. For example, clathrin-mediated endocytosis is proposed to be a dedicated system to traffic and process APP and is now being studied under the framework of AD biology. Furthermore, supporting the tau hypothesis, it has recently been proposed that tau may mediate neurotoxicity by altering the organization and dynamics of the actin cytoskeleton [90]. Similarly rhoA signaling was implicated in AD pathology, by its link to NGF (nerve growth factor) signaling.

**Table 2:** The top-10 canonical pathways associated with Neurological identified pathways identified in IPA (Ingenuity) to be significantly enriched with signature miRNA targets.

Ingenuity Canonical Pathways	−log(p-value)	Ratio	Molecules
Molecular Mechanisms of Cancer	1.79E01	6.07E-02	ADCY9,TGFBR1,ARHGEF12,PIK3R1,PTCH1,TAB2,SOS2, GNAQ,BMPR2,CRK,CCND1,BCL2L1, GNAI3,CCND2,CCND3, CBL, FOXO1 (includes EG:2308), IRS1,FZD6, SMAD4, PRKD3, RASA1, WNT1
Axonal Guidance Signaling	1.22E01	4.39E-02	EPHA7,ARHGEF12,BDNF,PIK3R1,PTCH1,SOS2,GNAQ,CRK, EIF4E, PDGFB,ROCK2,GNAI3,WASL, IGF1,CFL2,FZD6,PRKD3, RASA1, WNT1
PTEN Signaling	1.2E01	9.68E-02	BCL2L1,GHR,TGFBR1,CBL,FOXO1 (includes EG:2308),PIK3R1,FGFR1,SOS2,IGF1R,BMPR2,INSR,CCND1
Actin Cytoskeleton Signaling	1.08E01	5.88E-02	VAV2,ARHGEF12,PIK3R1,SOS2,RDX,CRK,PDGFB,F2,ROCK2, MYLK, WASL,CFL2,PPP1R12B, ARHGAP35
Huntington's Disease Signaling	1.06E01	5.88E-02	VTI1A,BDNF,HSPA1A/HSPA1B,PIK3R1,SOS2,GNAQ,CREB5, SNAP25, TAF9B,BCL2L1,IGF1,IGF1R, PRKD3,RASA1
Ephrin Receptor Signaling	9.58E00	6E-02	ROCK2,EPHA7,GNAI3,WASL,CFL2,SOS2,GNAQ,CRK,MAP4K4, CREB5, RASA1,PDGFB
IL-8 Signaling	9.55E00	6.22E-02	ROCK2,HMOX1,BCL2L1,GNAI3,NAPEPLD,CCND3,CCND2,PIK3R1, MAP4K4,PTGS2,PRKD3,CCND1
Cardiac Hypertrophy Signaling	9.28E00	5.31E-02	ADCY9,TGFBR1,CALM1 (includes others), PIK3R1,GNAQ,EIF4E, ROCK2, PLCD1,GNAI3, CACNA1E, IGF1, IRS1,IGF1R
Clathrin-mediated Endocytosis Signaling	9.27E00	6.15E-02	CD2AP,WASL,CBL,IGF1,SYNJ1,PIK3R1,USP9X,ITGB8,AAK1,CTTN, PDGFB,F2
RhoA Signaling	9.16E00	8.77E-02	ROCK2,MYLK,ARHGEF12,IGF1,CFL2,IGF1R,PPP1R12B,RDX,ARHGAP35,DLC1

The ratio represents the number of molecules identified as targets of at least 2 signature miRNAs divided by the total number of genes in the canonical pathway.

Next we applied an unbiased pathway enrichment analysis without any filter on pathway function (Table S6 in File SI). Interestingly, although not associated with neurology pathways directly, a canonical pathway linked to Type II Diabetes Mellitus signaling was identified to be significantly enriched for targets of the signature miRNAs. Amongst them included the insulin receptor gene (INSR), suppressor of cytokine signaling (SOCS), insulin receptor substrate 2 (IRS2) and tumor necrosis factor receptor (TNFR). There was a recent report that suggested a link with Insulin receptor signaling resistance and AD [91]. There was also evidence from multiple GWAS studies, that ApoE [92], Clu [93] and ABCA7 [94] gene polymorphisms were associated with AD and furthermore there was also biochemical data directly implicating lipid metabolism in both amyloid and tau pathology [95]. However, due to the mixed effects seen after statin treatment in AD clinical trials, the exact mechanism of lipid metabolism in AD pathogenesis remains to be established and our miRNA signature may point towards gene targets for potential future therapy linked to this pathway [96].

This plasma 7-miRNA profile represents one of the first global and non-invasive nucleic-acid based AD diagnostic assay, which can reliably predict with >95% accuracy whether a patient is suffering from AD. Furthermore, as a next step, it would be useful to obtain and test longitudinal plasma samples from a cohort of patients who have been diagnosed as MCI. Typically, ∼50% of MCI patients transition on to develop Alzheimer's and it would be insightful to determine if these miRNA biomarkers could identify the more susceptible group of patients. There was some evidence supporting the need of early detection biomarkers in the recent trial results released for two potential AD drugs, both targeting amyloid plaques. Bapineuzumab was developed jointly by Pfizer, Johnson and Johnson and Elan [97], while Solanezumab [98] was developed by Eli Lilly. Especially for Solanezumab, while the top line results suggested that there were no significant differences between patients on the drug or the placebo arm, there were some significant improvements in cognition and functioning in patients diagnosed with mild Alzheimer's (as opposed to no significant improvement for patients with moderate disease). This was recently presented at the 137th annual meeting of the American Neurological Association (Oct 7–9, 2012). Circulating blood biomarkers have the potential to positively impact patient comfort, ultimately leading to earlier diagnosis, which might enable patients to get on to treatments earlier and prevent this devastating disease.

## Supporting Information

Figure S1 **Scatter plots of selected miRNA differentiating Alzheimer and NC samples in Cohort 1.** Total RNA extracted from plasma samples was run using the nCounter assay on the Nanostring platform. Assay provided spike-in controls were used to account for lane-to-lane variation, followed by the top 100-miRNA expressers for content normalization. The normalized counts are represented on the Y-axis.
(TIF)

Figure S2 **Scatter plots of validated miRNAs differentiating Alzheimer and NC samples in Cohort 2.** Total RNA extracted from plasma samples was used for validating miRNA expression values using singleplex TaqMan assays. Ath-159a (spike-in) and hsa-miR-106a (endogenous) was used for normalization. All values were then normalized relative to the average of the 20 Control samples and plotted on the Y-axis.
(TIF)

File S1 **Contains Tables S1, S2, S3, S4, S5 and S6, with the following legends:** Table S1: Sample details from Cohort 1 samples. Table S2: Sample details from Cohort 2 samples. Table S3: miRNA sequence and ID used in this study based on miRbase v19 (Aug, 2012). Table S4: Differential expression of validated signature miRNAs for AD and NC samples (cohort 1 and cohort 2; TaqMan) using hsa-miR-106a/ath-159a or only ath-159a for normalization. Table S5: Performance characteristics of individual and signature miRNAs in predicting disease status in cohort 2 patients. Table S6: List of pathways enriched with genes targeted by at least 2 signature miRNAs. No pathway filters were set for this analysis.
(DOCX)
