# Supplementary figures and images for: Circulating miRNA Biomarkers for Alzheimer's Disease
Source: PLoS One. 2013 Jul 29;8(7):e69807. doi: 10.1371/journal.pone.0069807 (PMC3726785; doi:10.1371/journal.pone.0069807)

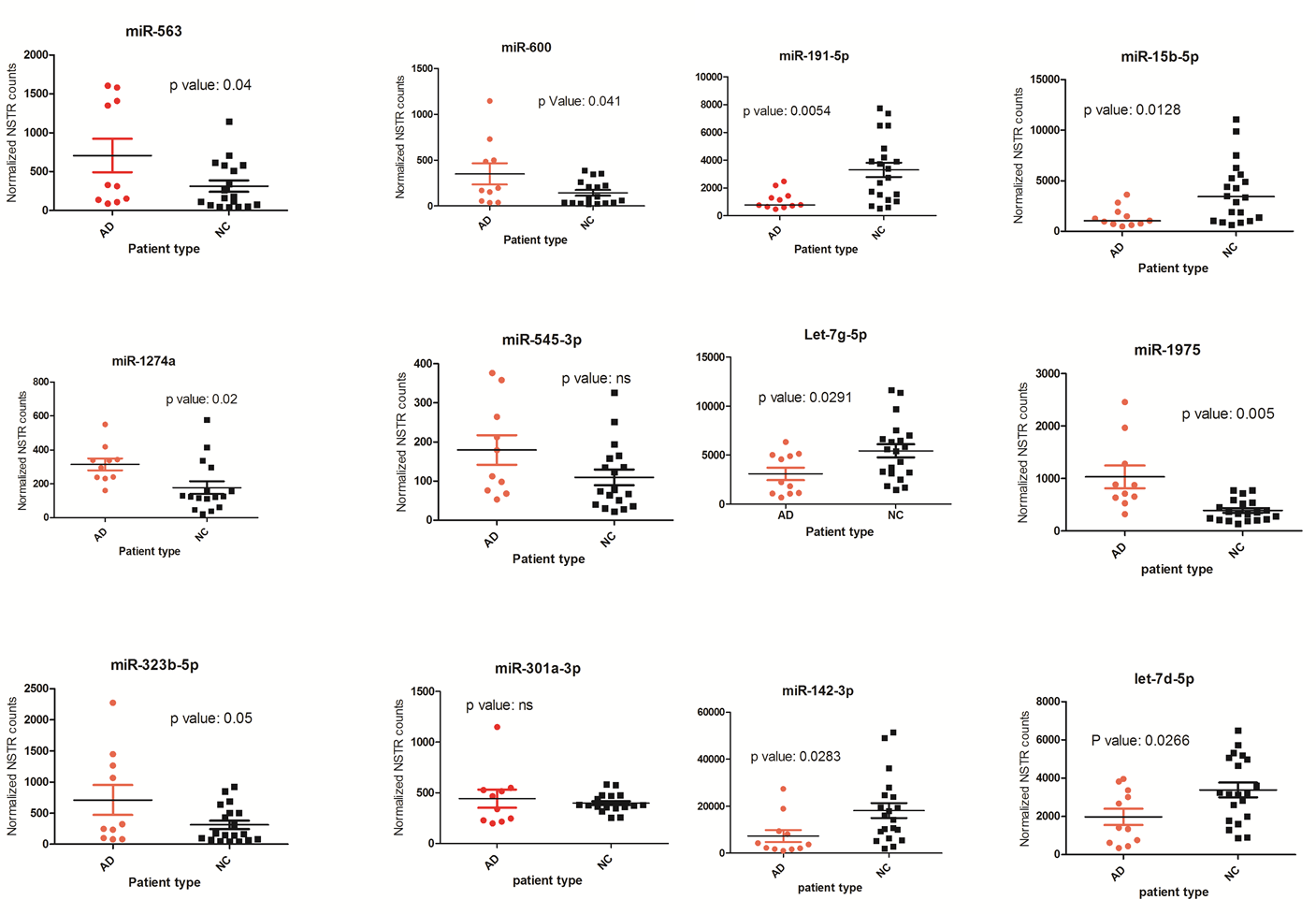

Supplement: Figure S1 — Scatter plots of selected miRNA differentiating Alzheimer and NC samples in Cohort 1. Total RNA extracted from plasma samples was run using the nCounter assay on the Nanostring platform. Assay provided spike-in controls were used to account for lane-to-lane variation, followed by the top 100-miRNA expressers for content normalization. The normalized counts are represented on the Y-axis. (TIF) [file pone.0069807.s001.tif]

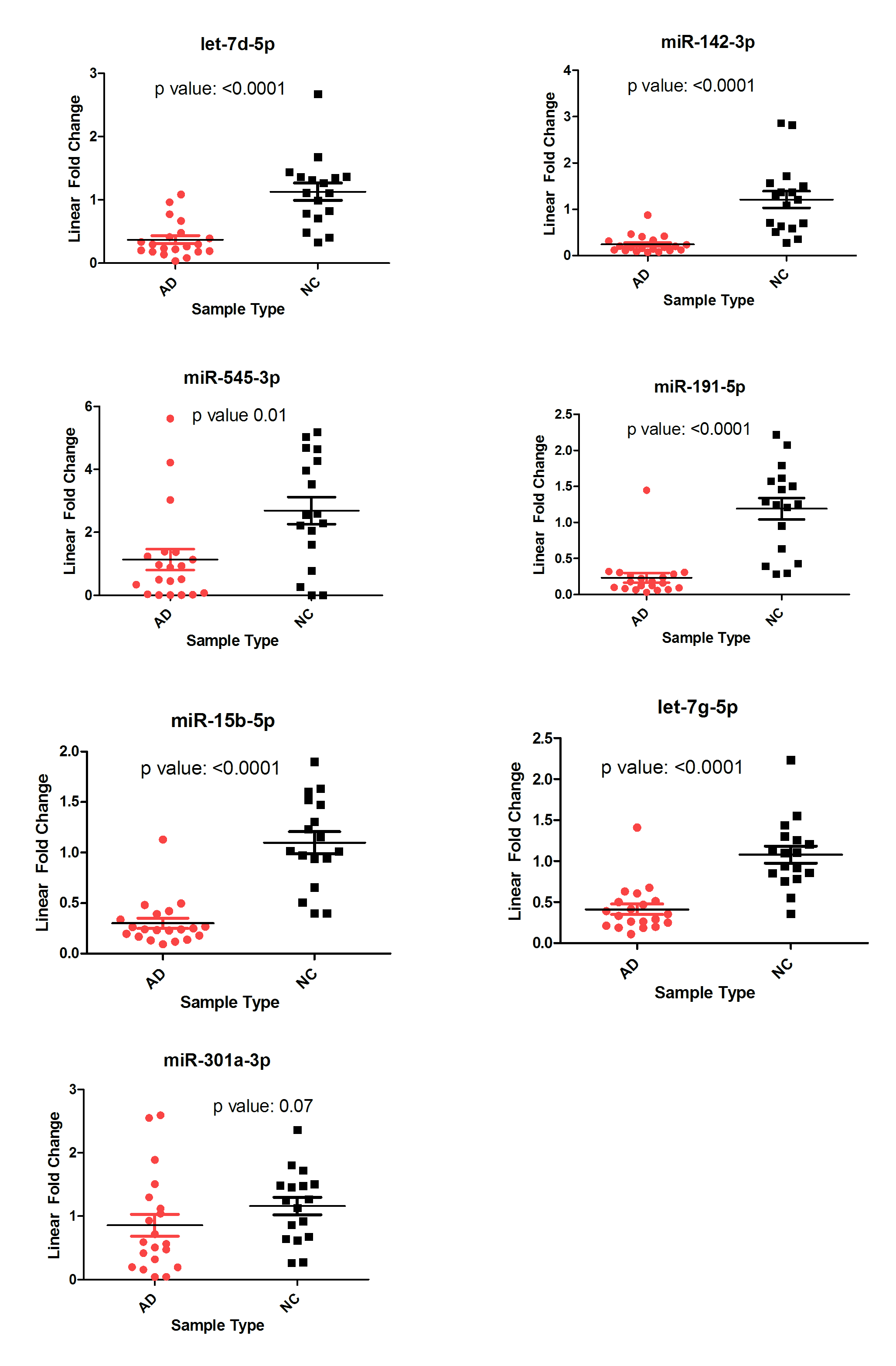

Supplement: Figure S2 — Scatter plots of validated miRNAs differentiating Alzheimer and NC samples in Cohort 2. Total RNA extracted from plasma samples was used for validating miRNA expression values using singleplex TaqMan assays. Ath-159a (spike-in) and hsa-miR-106a (endogenous) was used for normalization. All values were then normalized relative to the average of the 20 Control samples and plotted on the Y-axis. (TIF) [file pone.0069807.s002.tif]
