# Supplementary material for: Circulating miRNA Biomarkers for Alzheimer's Disease
Source: PLoS One. 2013 Jul 29;8(7):e69807. doi: 10.1371/journal.pone.0069807 (PMC3726785; doi:10.1371/journal.pone.0069807)
Supplement: File S1 — Contains Tables S1, S2, S3, S4, S5 and S6, with the following legends: Table S1: Sample details from Cohort 1 samples. Table S2: Sample details from Cohort 2 samples. Table S3: miRNA sequence and ID used in this study based on miRbase v19 (Aug, 2012). Table S4: Differential expression of validated signature miRNAs for AD and NC samples (cohort 1 and cohort 2; TaqMan) using hsa-miR-106a/ath-159a or only ath-159a for normalization. Table S5: Performance characteristics of individual and signature miRNAs in predicting disease status in cohort 2 patients. Table S6: List of pathways enriched with genes targeted by at least 2 signature miRNAs. No pathway filters were set for this analysis. (DOCX) [file pone.0069807.s003.docx]

**Table S1: Sample details from Cohort 1 samples**

| **Sample ID** | **Patient ID** | **Diagnosis** | **MMSE** | **Age** | **Gender** |
| --- | --- | --- | --- | --- | --- |
|  |  |  |  |  |  |
| 1 | 8001 | Alzheimers Disease (AD) | 17 | 83 | M |
| 2 | 8005 | Alzheimers Disease (AD) | 17 | 80 | M |
| 3 | 8006 | Alzheimers Disease (AD) | 22 | 91 | M |
| 4 | 8007 | Alzheimers Disease (AD) | 16 | 78 | F |
| 5 | 8011 | Alzheimers Disease (AD) | 23 | 74 | M |
| 9 | 8017 | Alzheimers Disease (AD) | 24 | 88 | M |
| 10 | 8019 | Alzheimers Disease (AD) | 18 | 76 | F |
| 12 | 8026 | Alzheimers Disease (AD) | 14 | 78 | F |
| 14 | 8029 | Alzheimers Disease (AD) | 15 | 74 | M |
| 19 | 8058 | Alzheimers Disease (AD) | 15 | 72 | F |
| 20 | 8061 | Alzheimers Disease (AD) | 16 | 82 | F |
| 6 | 8012 | mild cognitive impairment (MCI) | 23 | 82 | F |
| 7 | 8015 | mild cognitive impairment (MCI) | 23 | 80 | F |
| 8 | 8016 | mild cognitive impairment (MCI) | 27 | 79 | F |
| 11 | 8023 | mild cognitive impairment (MCI) | 26 | 63 | F |
| 13 | 8028 | mild cognitive impairment (MCI) | 23 | 79 | F |
| 15 | 8031 | mild cognitive impairment (MCI) | 25 | 80 | M |
| 16 | 8033 | mild cognitive impairment (MCI) | 26 | 74 | F |
| 17 | 8036 | mild cognitive impairment (MCI) | 26 | 84 | M |
| 18 | 8039 | mild cognitive impairment (MCI) | 23 | 76 | M |
| 23 | 7090022 | NC | 27 | 60 | M |
| 21 | 7090003 | NC | 30 | 60 | F |
| 25 | 7090033 | NC | 30.0 | 68 | F |
| 26 | 7090040 | NC | 30.0 | 60 | M |
| 27 | 7090045 | NC | 30.0 | 64 | M |
| 28 | 7090055 | NC | 27.0 | 77 | M |
| 29 | 7090076 | NC | 30.0 | 69 | M |
| 30 | 7090082 | NC | 30.0 | 60 | F |
| 31 | 7090084 | NC | 30.0 | 72 | M |
| 32 | 7090085 | NC | 30.0 | 66 | F |
| 33 | 7090087 | NC | 30.0 | 71 | M |
| 34 | 7090088 | NC | 29.0 | 60 | F |
| 35 | 7090089 | NC | 30.0 | 61 | M |
| 36 | 7090090 | NC | 30.0 | 69 | M |
| 37 | 7090091 | NC | 30.0 | 74 | F |
| 38 | 7090092 | NC | 30.0 | 71 | F |
| 39 | 7090094 | NC | 27.0 | 63 | M |
| 40 | 7090095 | NC | 30.0 | 74 | F |

**Table S2: Sample details from Cohort 2 samples**

| **Sample ID** | **Patient ID** | **Diagnosis** | **MMSE** | **Age** | **Gender** |
| --- | --- | --- | --- | --- | --- |
|  |  |  |  |  |  |
| 1 | 8037 | Alzheimers Disease (AD) | 14 | 78 | F |
| 2 | 8038 | Alzheimers Disease (AD) | 15 | 76 | M |
| 3 | 8056 | Alzheimers Disease (AD) | 17 | 75 | M |
| 4 | 8057 | Alzheimers Disease (AD) | 13 | 76 | F |
| 5 | 8072 | Alzheimers Disease (AD) | 22 | 61 | M |
| 6 | 8076 | Alzheimers Disease (AD) | 18 | 70 | F |
| 7 | 8077 | Alzheimers Disease (AD) | 9 | 77 | F |
| 8 | 8086 | Alzheimers Disease (AD) | 21 | 66 | F |
| 9 | 8089 | Alzheimers Disease (AD) | 18 | 67 | F |
| 10 | 8092 | Alzheimers Disease (AD) | 15 | 70 | F |
| 11 | 8106 | Alzheimers Disease (AD) | 6 | 71 | F |
| 12 | 8107 | Alzheimers Disease (AD) | 18 | 74 | M |
| 13 | 8118 | Alzheimers Disease (AD) | 17 | 72 | M |
| 14 | 8122 | Alzheimers Disease (AD) | 18 | 69 | F |
| 15 | 8129 | Alzheimers Disease (AD) | 18 | 76 | M |
| 16 | 8133 | Alzheimers Disease (AD) | 15 | 62 | M |
| 17 | 8192 | Alzheimers Disease (AD) | 20 | 63 | M |
| 18 | 8200 | Alzheimers Disease (AD) | 22 | 59 | F |
| 19 | 8207 | Alzheimers Disease (AD) | 18 | 62 | M |
| 37 | 8133 | Alzheimers Disease (AD) | 16 | 62 | M |
| 20 | 7090031 | NC | 30 | 62 | M |
| 21 | 7090050 | NC | 30 | 63 | F |
| 22 | 7090068 | NC | 30 | 66 | M |
| 23 | 7090072 | NC | 30 | 64 | F |
| 24 | 7090074 | NC | 29 | 62 | F |
| 25 | 7090075 | NC | 30 | 75 | F |
| 26 | 7090079 | NC | 24 | 75 | M |
| 27 | 7090081 | NC | 30 | 63 | F |
| 28 | 7090082 | NC | 30 | 60 | F |
| 29 | 7090083 | NC | 30 | 69 | M |
| 30 | 7090086 | NC | 30 | 68 | M |
| 31 | 7090093 | NC | 30 | 66 | M |
| 32 | 7090097 | NC | 30 | 64 | F |
| 33 | 7090098 | NC | 30 | 60 | F |
| 34 | 70057518 | NC | 30 | 63 | M |
| 35 | 70057524 | NC | 29 | 67 | M |
| 36 | 70057529 | NC | 30 | 68 | M |

**Table S3: miRNA sequence and ID used in this study based on miRbase v19 (Aug, 2012)**

| **miRNA Name** | **miRBASE#** | **Sequence** |
| --- | --- | --- |
|  |  |  |
| hsa-miR-142-3p | MIMAT0000434 | UGUAGUGUUUCCUACUUUAUGGA |
| hsa-miR-545-3p | MIMAT0003165 | UCAGCAAACAUUUAUUGUGUGC |
| hsa-let-7d-5p | MIMAT0000065 | AGAGGUAGUAGGUUGCAUAGUU |
| hsa-miR-191-5p | MIMAT0000440 | CAACGGAAUCCCAAAAGCAGCUG |
| hsa-miR-301a-3p | MIMAT0000688 | CAGUGCAAUAGUAUUGUCAAAGC |
| hsa-let-7g-5p | MIMAT0000414 | UGAGGUAGUAGUUUGUACAGUU |
| hsa-miR-15b-5p | MIMAT0000417 | UAGCAGCACAUCAUGGUUUACA |
| hsa-miR-106a-5p | MIMAT0000103 | AAAAGUGCUUACAGUGCAGGUAG |
| ath-159a | MIMAT0000177 | UUUGGAUUGAAGGGAGCUCUA |
| hsa- miR -323b-5p | [MIMAT0001630](http://www.mirbase.org/cgi-bin/mature.pl?mature_acc=MIMAT0001630) | AGGUUGUCCGUGGUGAGUUCGCA |
| hsa- miR -563 | [MIMAT0003227](http://www.mirbase.org/cgi-bin/mature.pl?mature_acc=MIMAT0003227) | AGGUUGACAUACGUUUCCC |
| hsa- miR -600 | [MIMAT0003268](http://www.mirbase.org/cgi-bin/mature.pl?mature_acc=MIMAT0003268) | ACUUACAGACAAGAGCCUUGCUC |
| hsa- miR -1274a | Dead entry | fragment of a Lys tRNA; PMID: 20818168 |
| hsa- miR -1975 | Dead entry | fragment of Y RNA; PMID: 20483914 |

| miRNA name | TaqMan Cohort 1(miR-106a and ath-159a) | | Taqman Cohort 1 (ath-159a only) | | TaqMan Cohort 2 (miR-106a and ath-159a) | | Taqman Cohort 2 (ath-159a only) | |
| --- | --- | --- | --- | --- | --- | --- | --- | --- |
|  | Fold Change | P value | Fold Change | P value | Fold Change | P value | Fold change | P value |
| hsa-let-7d | 3.01 | 0.0001 | 7.34 | 0.0001 | 3.03 | <0.0001 | 4.73 | <0.0001 |
| hsa-let-7g | 2.26 | 0.001 | 6.22 | 0.0003 | 2.62 | <0.0001 | 3.92 | <0.0001 |
| hsa-miR-15b | 3.45 | 0.001 | 8.75 | 0.0005 | 3.65 | <0.0001 | 5.62 | <0.0001 |
| hsa-miR-142-3p | 3.84 | 0.0001 | 8.74 | 0.0002 | 5.04 | <0.0001 | 7.98 | <0.0001 |
| hsa-miR-191 | 3.38 | 0.002 | 8.4 | 0.001 | 5.15 | <0.0001 | 6.76 | <0.0001 |
| hsa-miR-301a | 2.98 | 0.0006 | 7.27 | 0.0004 | 1.35 | 0.07 | 2.57 | 0.002 |
| hsa-miR-545 | 2.49 | 0.03 | 5.78 | 0.009 | 2.37 | 0.01 | 3.07 | 0.001 |

**Table S4: Differential expression of validated signature miRNAs for AD and NC samples (cohort 1 and cohort 2; TaqMan) using hsa-miR-106a/ath-159a or only ath-159a for normalization.**

**Table S5: Performance characteristics of individual and signature miRNAs in predicting disease status in cohort 2 patients.**

| **miRNA signature** | **Sensitivity** | | **Specificity** | **AUC** |
| --- | --- | --- | --- | --- |
|  | | | | |
| **Individual miRNAs** | | | | |
|  | | | | |
| miR.15b | 0.85 | | 0.882353 | 0.956 |
| miR.142.3p | 0.65 | | 1 | 0.956 |
| miR.191 | 0.95 | | 0.7647059 | 0.953 |
| let.7g | 0.95 | | 0.5294118 | 0.926 |
| let.7d | 0.75 | | 0.882353 | 0.924 |
| miR.545 | 0.2 | | 0.882353 | 0.741 |
| miR.301 | 0.25 | | 1 | 0.674 |
|  | | | | |
| **Signature miRNAs** | | | | |
|  | |  |  |  |
| miR.545 miR.15b | | 0.9 | 0.9411765 | 0.965 |
| miR.301 miR.545 let.7g miR.15b | | 0.95 | 0.882353 | 0.965 |
| miR.191 miR.15b | | 0.95 | 0.8235294 | 0.962 |
| miR.191 miR.301 miR.545 | | 0.95 | 0.8235294 | 0.956 |
| miR.191 miR.301 miR.545 miR.15b | | 0.95 | 0.8235294 | 0.956 |
| miR.545 let.7g miR.15b | | 0.95 | 0.9411765 | 0.953 |
| let.7g miR.15b | | 0.95 | 0.8235294 | 0.941 |
| miR.301 let.7g miR.15b | | 0.95 | 0.8235294 | 0.932 |

**Table S6: List of pathways enriched with genes targeted by at least 2 signature miRNAs. No pathway filters were set for this analysis.**

| **Pathways** | **-Log(p values)** |
| --- | --- |
| PPARÎ±/RXRÎ± Activation | 6.11E+00 |
| Molecular Mechanisms of Cancer | 4.85E+00 |
| Glioblastoma Multiforme Signaling | 4.42E+00 |
| Factors Promoting Cardiogenesis in Vertebrates | 4.20E+00 |
| Wnt/Î²-catenin Signaling | 3.70E+00 |
| HER-2 Signaling in Breast Cancer | 3.32E+00 |
| Cell Cycle: G1/S Checkpoint Regulation | 3.31E+00 |
| Type II Diabetes Mellitus Signaling | 3.10E+00 |
| GADD45 Signaling | 2.65E+00 |
| Cyclins and Cell Cycle Regulation | 2.57E+00 |
| Calcium Transport I | 2.55E+00 |
| PTEN Signaling | 2.47E+00 |
| TGF-Î² Signaling | 2.44E+00 |
| Salvage Pathways of Pyrimidine Ribonucleotides | 2.40E+00 |
| Estrogen-mediated S-phase Entry | 2.26E+00 |
| D-myo-inositol-5-phosphate Metabolism | 2.21E+00 |
| Role of Osteoblasts, Osteoclasts and Chondrocytes in Rheumatoid Arthritis | 2.18E+00 |
| 3-phosphoinositide Degradation | 2.11E+00 |
| Cardiac Hypertrophy Signaling | 2.11E+00 |
